# In Very Young Infants Severity of Acute Bronchiolitis Depends On Carried Viruses

**DOI:** 10.1371/journal.pone.0004596

**Published:** 2009-02-25

**Authors:** Christophe Marguet, Marc Lubrano, Marie Gueudin, Pascal Le Roux, Antoine Deschildre, Chantal Forget, Laure Couderc, Daniel Siret, Marie-Dominique Donnou, Michael Bubenheim, Astrid Vabret, François Freymuth

**Affiliations:** 1 Respiratory Diseases, Allergy and CF Unit, Pediatric Department, Rouen University Hospital Charles Nicolle, Rouen, France; 2 Department of Virology, Rouen University Hospital Charles Nicolle, Rouen, France; 3 Respiratory Diseases, Allergy and CF Unit, Pediatric Department, Regional Hospital-Flaubert, Le Havre, France; 4 Respiratory Diseases, Allergy and CF Unit, Pediatric Department, Lille University Hospital Jeanne de Flandres, Lille, France; 5 Respiratory Diseases and Allergy Unit, Pediatric Department, Regional Hospital, Elbeuf, France; 6 Respiratory Diseases, Allergy and CF Unit, Pediatric Department, Regional Hospital Moulin du Pré, Saint-Nazaire, France; 7 Pediatric Lung Function Tests and Respiratory Diseases Unit, Brest University Hospital, Morvan, Brest, France; 8 Biostatistics Department, Rouen University Hospital-Charles Nicolle, Rouen, France; 9 Laboratory of Human and Molecular Virology, Caen University Hospital Clemenceau, Caen, France; University of Giessen Lung Center, Germany

## Abstract

**Background:**

RT amplification reaction has revealed that various single viruses or viral co-infections caused acute bronchiolitis in infants, and RV appeared to have a growing involvement in early respiratory diseases. Because remaining controversial, the objective was to determine prospectively the respective role of RSV, RV, hMPV and co-infections on the severity of acute bronchiolitis in very young infants.

**Methods and Principal Findings:**

209 infants (median age: 2.4 months) were enrolled in a prospective study of infants <1 year old, hospitalized for a first episode of bronchiolitis during the winter epidemic season and with no high risk for severe disease. The severity was assessed by recording SaO_2_% at admission, a daily clinical score (scale 0–18), the duration of oxygen supplementation and the length of hospitalization. Viruses were identified in 94.7% by RT amplification reaction: RSV only (45.8%), RV only (7.2%), hMPV only (3.8%), dual RSV/RV (14.3%), and other virus only (2%) or coinfections (9%). RV compared respectively with RSV and dual RSV/RV infection caused a significant less severe disease with a lower clinical score (5[3.2–6] vs. 6[4–8], p = 0.01 and 5.5[5–7], p = 0.04), a shorter time in oxygen supplementation (0[0–1] days vs. 2[0–3] days, p = 0.02 and 2[0–3] days, p = 0.03) and a shorter hospital stay (3[3–4.7] days vs.6 [5–8] days, p = 0.001 and 5[4–6] days, p = 0.04). Conversely, RSV infants had also longer duration of hospitalization in comparison with RSV/RV (p = 0.01) and hMPV (p = 0.04). The multivariate analyses showed that the type of virus carried was independently associated with the duration of hospitalization.

**Conclusion:**

This study underlined the role of RV in early respiratory diseases, as frequently carried by young infants with a first acute bronchiolitis. RSV caused the more severe disease and conversely RV the lesser severity. No additional effect of dual RSV/RV infection was observed on the severity.

## Introduction

Acute bronchiolitis is the most common respiratory disease in infants under 2 years of age. Although most infants recover within five days, annual bronchiolitis hospitalization among infants younger than 1 year has been assessed at 31.2/1000 [1]. During the epidemic season, up to 90% of this acute wheezing disease has been attributed to respiratory syncytial virus (RSV). However, the recent use of RT amplification reaction has modified our knowledge as regards the epidemiology during the winter epidemic season, revealing the role of other viruses such as rhinovirus (RV) and human metapneumovirus (hMPV), and at a less frequency enterovirus, coronavirus or bocavirus [2]. Moreover, dual viral infection appeared more frequently, estimated from 20% to 30% [3]–[5]. Accompanying the change in epidemiology, the question was raised concerning severity as regards these various carried viruses. The results have remained controversial. For some authors, the severity was similar between RSV and RV [6], RSV and hMPV bronchiolitis [4]. However, Korppi *et al.* [6] defended the fact that RSV might be responsible for a more severe disease, as this virus concerned younger infants [7]. In comparison with RSV, RV has also been associated with either a more severe bronchiolitis [3], or a shorter duration of the disease [8]. In one study, hMPV caused more moderate bronchiolitis than those related to RSV [9]. The involvement of viral co-infections in the expression of the severity of bronchiolitis was also debated. In fact, dual RSV/hMPV or RSV/RV was shown to worsen the clinical severity and increase the duration of hospitalization [10]–[12]. Conversely, Xepapadaki *et al* [4] and Papadopoulos *et al.* [3] did not confirm the aggravation effect of these dual infections. Recently, the fact that carrying at least any two viruses augmented the risk to be transferred to PICU has been reported [5]. The aim of this prospective study was to assess the clinical severity and viral etiology in infants hospitalized with a first episode of acute bronchiolitis during the epidemic season. Within this very young population, RSV caused more marked severe bronchiolitis than did RV, and RV co-infection but neither aggravated the clinical features nor the length of the hospitalization. HMPV related bronchiolitis were more frequently associated with hypoxemia at admission.

### Study design, patients and methods

#### Ethics statement

This study was conducted according to the principles expressed in the Declaration of Helsinki. The French North West I Hospital Ethics Committee (*Comité de Protection des Personnes “Nord-Ouest I”*, *University Hospital*, *Rouen*, *France*) approved the research study. All patients provided written informed consent for the collection of samples and subsequent analysis.

A prospective multicentre study was conducted in infants hospitalized for a first episode of acute bronchiolitis during three epidemic seasons (November to March 2002–2004). Four pediatric centers in a same geographical location (North West of France) participated in the study. Inclusion criteria were as follows: Infants aged from 1 month to 1 year, requiring hospitalization for a first episode of acute bronchiolitis. The diagnosis was based on dyspnea and wheezing or crackles associated with lung hyperinflation on chest X-ray. Exclusion criteria were any administration of steroids, history of wheezing, or underlying disease susceptible to modify the natural outcome (immunodeficiency, chronic lung disease, cystic fibrosis, congenital heart disease etc.). Medical history and demographic data were collected. Atopy was defined by asthma, allergic rhinitis or eczema in first degree relatives, or by eczema in infants. Diagnosis and severity of bronchiolitis were first established in the Pediatric Emergency Department, where the decision to be hospitalized was made. The severity of the disease was assessed with SaO_2_%, a clinical score, the duration of oxygen requirement, and the length of hospitalization. The clinical score associated standard criteria previously described [13], [14] (Table 1). Each item was scaled from 0 to 1 and the score varied from 0 to 18. Because the disease could worsen after admission, the infant was further scored daily. The highest score observed during the hospitalization was retained for determining the severity of the disease. The discharge of the patient was based on the following criteria: SaO_2_%≥95%, normal respiratory rate (RR≤47/min for infants aged <2 mo, and ≤42/min when older) and no alteration in feeding during the past 24 h nor other complications directly related to the bronchiolitis. The length of the hospitalization was calculated as the time between the admission and the validation of the discharge criteria. Any prolonged hospitalization for other reasons was therefore not taken into account. The Rouen Hospital Ethics Committee approved the research study and informed consent was obtained from the parents.

**Table 1:** clinical score.

	0	1 = mild	2 = marked
Intercostals retraction			
Xiphoid retraction			
Chest movement synchronization			
Nasal flaring			
**Score I = **			
	0	1	2
Wheezing	None	E	E+I or no more sounds
I/E ratio	I>E	I = E	I<E
Reactivity	Good	Tired	Poor
Feeding	Unchanged	enteral	parenteral
Lung hyperinflation Chest X-ray	No	Yes with CTR≥0.5	Yes with CTR<0.5
**Score II = **			
**final score = Scores I+II**			

E = expiratory; I = inspiratory; CTR = cardiothoracic ratio.

* Chest X-ray was assessed once at the admission.

### Detection of viruses

Antigens for RSV, influenza virus A and B, parainfluenza type 1, 2, and 3, and adenovirus were detected by direct immunofluorescence assay (DFA) from nasopharyngeal aspiration (NPA) samples, employing commercial monoclonal antibodies conjugated with fluorescein isothiocyanate (IMAGEN, Dako, Denmark) [15]. A positive result was indicated by DFA, and to the finding of at least one cell showing a typical fluorescence pattern, provided that at least 20 respiratory cells were available in the sample. All the samples were further analyzed by using molecular methods including four multiplex RT-PCR, as previously described [2], and an adenovirus PCR (Adenovirus Consensus®, Argene). The multiplex PCR targeted 14 respiratory viruses: RSV, influenza viruses A, B and C, hMPV, parainfluenza viruses (PIV) types 1, 2, 3, 4, RV, enterovirus (EV), human coronavirus OC43, 229E and NL63. The molecular methods allowed us to confirm the presence of the viruses which were detected by the DFA, and to find viruses that were not included in the DFA.

### Statistical analysis

Pairwise correlation among the SaO_2_%, clinical score, length of oxygen supply, demographic data and hospitalization were assessed using Spearman's rank test (Rho). For nominal variables, namely virus groups, a Kruskall-Wallis or a Mann–Whitney's test were also used. Univariate analyses were performed to assess the association between these variables and severity criteria. Severity was then analyzed as nominal variables using for clinical score, length of oxygen supplementation and hospitalization the 50^th^ percentile, and for SaO2% the significant hypoxemia threshold of 92%. Multivariate analyses were performed by using stepwise logistic regression to select variables independently associated with the virus-related severity. A Chi-2 test, and Fischer's test if required, was applied for the qualitative analyses. In all the analyses, a value of p<0.05 was considered statistically significant. The data were displayed as median and 25^th^–75^th^ quartiles. The statistical analyses were performed using Statview 5.0 (SAS Institute Inc., Cary-NC CA).

## Results

Two hundred nine infants were included. The median age was 2.4[1.6–3.9] months, range (1–10.5), of which 91%<6 months, and the sex ratio was 1.3. A total of 94.7% infants carried at least one virus (Table 2), 93.2% of RSV-associated bronchiolitis were detected by IF and the remaining 6.7% by RT-PCR. Twenty-one (10%) infants had a severe bronchiolitis with a clinical score >7, 96 (46.9%) required oxygen supplementation, seven were transferred to PICU and four intubated.

**Table 2:** characteristics of the population studied.

N = 209	*n*	%
Boys	118	57.5
Familial history of atopy	142	67.9
Personal history of atopy	48	23.0
Premature term >34 weeks *	12	5.7
Passive tobacco	128	61.2
Causes of hospitalization		
Severity	135	64.6
Age <3 months	126	60.3
Geographical care access	22	10.5
Socioeconomic difficulties	13	6.2
Identified viruses		94.7
RSV	134	64.1
RV	56	26.8
HMPV	16	7.6
Other**	16	7.6
Viral etiology		
RSV alone	96	45.8
RV alone	15	7.2
HMPV alone	8	3.8
Other virus alone***	4	2.0
Dual RSV/RV infection	30	14.3
Dual RSV/hMPV infection	3	1.4
Dual hMPV/RV infection	5	2.4
Other co-infections****	11	5.2
Severity	median [25^th^–75^th^]	
Clinical Score	5[4–7]	
%SaO_2_	95[92–97]	
Oxygen supplementation (days)	1[0–3]	
Length of hospitalization (days)	5[3.8–7]	

* Gestational age <34 weeks was an exclusion criteria.

** Enterovirus [EV] (n = 9), coronavirus (n = 2), influenza A virus [MIA] (n = 1), parainfluenza virus [PIV 1+] (n = 2) or PIV 3 (n = 2).

*** MIA (n = 1), PIV 1 (n = 2) or PIV 3 (n = 1).

**** RSV/coronavirus (n = 2), RSV/EV (n = 2), RSV/RV/EV (n = 5), RSV/RV/MIA(n = 1).

### Severity and infant characteristics

The clinical score, length of oxygen supplementation and duration of hospitalization were significantly correlated to each other: score and duration of oxygen supplementation (Rho = 0.482, p<0.0001); score and length of hospitalization (Rho = 0.393, p<0.0001); duration of oxygen supplementation and hospitalization (Rho = 0.527, p<0.0001). The age was inversely correlated with the length of hospitalization (Rho = −0.296, p<0.0001). The girls had an higher clinical score than the boys: 6 [4–8] vs. 5 [4–7] p<0.036; the infants with a history of family atopy had a lower clinical score (5 [4–7] vs. 7[5–9], p = 0.002) and required a shorter time of oxygen supplementation (2[0–4]d vs. 1[0–3]d, p = 0.009). Passive tobacco exposure was associated with a longer duration of hospitalization: 5[4–8] vs. 5 [3–6], p = 0.004.

### Severity by type of virus

The dichotomous analysis by identified virus is displayed in Table 3. The univariate logistic regression showed that to carry RSV increased the risk to have a clinical score >6 (OR = 2.4[1.1–4.3]95%CI), oxygen supplementation (OR = 2.5[1.28–4.97] 95%CI) and hospitalization>5 d (OR = 6.2[2.99–12.80]95%CI). To carry RV decreased the risk to be hospitalized>5 d (OR = 0.43[0.23–0.83]95%CI) and hypoxemic at the admission (OR = 0.17[0.13–0.99]95%CI). The presence of hMPV lowered the risk to be hospitalized>5 d (OR = 0.33[0.11–0.96] 95%CI) and increased the risk to have SaO_2_%≤92% at the admission (OR = 5.88[1.98–16.9]95%CI).

**Table 3:** Severity and, RSV and RV or hMPV virus carriage.

Severity	RSV	RSV	p*	RV	RV	p*	HMPV	HMPV	p*
	+ve	−ve		+ve	−ve		+ve	−ve	
	*n* = 134	*n* = 47		*n* = 56	*n* = 125		*n* = 16	*n* = 165	
Age (months)	2.4[1.5–3.6]	2.3[1.6–5.0]	-	2.4[1.6–4.3]	2.41.6–3.6]	-	2.6[1.7–5.5]	2.4[1.5–3.6]	-
Clinical score	6[4–7]	5[3–6]	0.004	5[4–6.5]	5.5[4–7]	-	5[3.5–7]	5[4–7]	-
≤6 (%)	18.1	66	0.04	57.1	50	0.01	62.5	51.2	0.056
>6 (%)	81.9	34		42.9	50		37.5	48.7	
O_2_ (days)	2[0–3]	0[0–2]	0.004	1[0–3]	1[0–3]	-	0.5[0–4]	1[0–3]	-
No (%)	34.7	57.5	0.009	53.6	60.8	-	50	59.3	-
Yes (%)	65.3	42.5		46.4	39.2		50	40.6	
%SaO_2_	95[92–97]	95[92–96.2]	-	95[93–96]	95[92–97]	-	91.5[90–95.5]	95[93–97]	0.008
≤92% (%)	18.1	17.8	-	9	21.7	0.056	50	14.7	0.002
>92% (%)	81.9	82.2		91	78.2		50	85.3	
Hospitalization (days)	6[4–8]	4[3–5]	<0.0001	4[3–6]	6[4–8]	0.005	4[3–7.5]	5[4–7]	-
≤5 (%)	39.2	70.2	<0.001	51.8	32	0.01	46.4	35.7	-
>5 (%)	67	29.7		48.9	68		53.6	64.2	

• Kruskall-Wallis non parametric test. Results are expressed in median[25^th^–75^th^]quartiles or in percentage, as indicated in the table.

### Severity and single or dual viral infection

**Figure 1:**
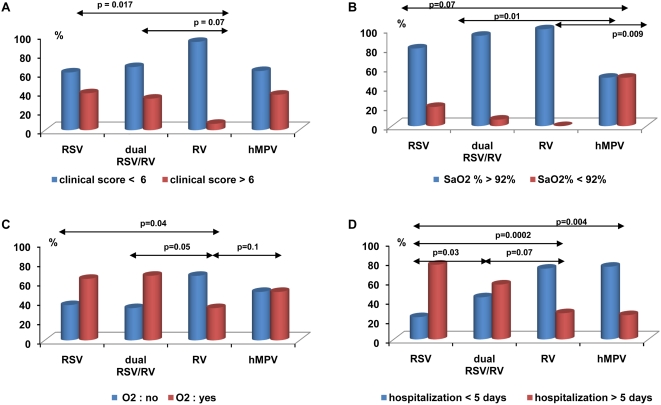
Severity of acute bronchiolitis with regards to the viral etiology. The following groups were compared: RVS alone (n = 96), RV alone (n = 15), dual RVS/RV infection (n = 30), and hMPV alone (n = 8). The studied criteria of severity were clinical score (A), SaO_2_% at the admission (B), oxygen supplementation (C) and duration of hospitalization (D).

The large variety of viruses and viral co-infections found restricted the analyses to RSV alone, RV alone, dual RSV/RV infection, and hMPV alone, the other groups being too small (Table 2). For this subpopulation, the clinical score was 6[4–7], SaO_2_% was 95[92.5–97]% length of O_2_ supplementation and hospitalization were 1[0–3] day and 5[4–7] days, respectively. There was a significant difference in the severity between RV, RSV, RV/RSV and hMPV as shown in Table 4 and Figure 1. Compared to RSV, infants with RV had a lower risk to present a severe disease as regards being supplied with oxygen (OR = 0.29[0.09–0.90]95%CI) and to stay >5 days (OR = 0.11[0.03–0.37]95%CI). This was also observed for hMPV with a lower risk to be hospitalized>5 days (OR = 0.09[0.019–0.53]95%CI). Conversely, the risk to have SaO_2_%≤92% at admission tended to increase in hMPV compared with RSV and RSV/RV bronchiolitis: OR = 4[1.0–16.7]95%CI and OR = 14.2[1.92–100]95%CI, respectively. All the infants suffering from RV bronchiolitis had SaO2%>92%, preventing the calculation of odds ratio. The multivariate analyses including the need for oxygen, clinical score, SaO_2_% and age showed that only the risk to stay <5 days decreased in RSV/RV (OR = 0.26[0.09–0.76]95%CI), RV (OR = 0.13[0.03–0.57]95%CI) and hMPV (OR = 0.09[0.01–0.69] 95%CI) compared with RSV bronchiolitis.

**Table 4:** Severity and single or dual viral infections.

Severity (unit)	RSV	RVS/RV	RV	hMPV	*p* *
	*n* = 96	*n* = 30	*n* = 15	*n* = 8	
**Age (months)**	2.2 1.5–3.4]	2.7 1.8–3.6]	1.7[1.4–4.8]	2.6[2.2–5.4]	-
**Birth weight (g)**	3290 [2952–3592]	3375 [3000–3700]	3280 [2832–3585]	3230 [3000–3605]	-
**Boys (%)**	60.4	50.7	40	50	-
**Family atopy (%)**	65.6	63.3	80	62.5	-
**Personal atopy (%)**	22.9	30	13.3	62.5†‡	0.08
**Passive tobacco exposure (%)**	63.5	46.7	53.3	62.5	-
**Onset of symptoms (days)**					
**Rhinitis**	2[2–4]	3[1.2–5.7]	1.5[1–3]**^§^**	6.5[2–11]**^†^**	0.04
**Cough**	2[1–4]	3[2–5]	3[2–4.7] **^#^**	5[2–10]^*^	0.08
**Dyspnea**	1[0.5–2]	1[0.7–2.2]	1.5[1–3]	2[1–2]	-
**Wheezing**	1[0–2]	1[0–2]	2[1–4.5]**^§^**	1[0.5–2]	0.13
**Clinical score**	6 [4–8]	5.5 [5–7]	5[3.2–6]**^§#^**	5.5[3.5–8]	0.12
**Duration of O_2_ supplementation (days)**	2[0–3]	2[0–3]	0[0–1] **^§#^**	0.5[0–2.5]	0.13
**%SaO_2_**	95[92–97]	94[93–96]	96[95–97]	92.5[89–95.5]^*‡^	0.14
**Length of hospitalization (days)**	6 [5–8]	5 [4–6]**^¥^**	3 [3–4.7] **^§#^**	3.5[2.5–4.5]**^†^**	0.0002

Results are expressed in median [25^th^–75^th^] quartiles. ^*^Kruskall-Wallis non parametric test. p<0.05: ^†^hMPV vs. RSV; ^‡^hMPV vs. RV; ^§^RV vs. RSV; ^¥^RVS/RV vs. RSV; ^#^RV vs. RSV/RV; p<0.1. ^*^hMPV vs. RSV.

## Discussion

The availability of multiplex RT amplification reactions has modified our knowledge as regards the viral epidemiology of the winter bronchiolitis, where RSV has for a long time been considered the unique etiologic agent. It is now well established that rhinoviruses and human metapneumoviruses have also been involved in infantile bronchiolitis. However, the phenotypes as regards these new viruses should to be clarified, as the available data still remain controversial. In the present study, by analyzing various severity criteria, we showed that RSV remained the most aggressive agent in young infants, and conversely RV caused less severe bronchiolitis. Furthermore, dual RSV/RV infection did not aggravate the severity of illness, tending to be more moderate than RSV related bronchiolitis. In addition, hMPV bronchiolitis has been distinguished by a more marked hypoxemia at the admission.

The use of molecular amplification reaction was expected to achieve a better viral detection, which ranged from 70.5% to 96.1% in comparable studies [2], [3], [5], [16], [17]. In agreement with our previous reported experience [2], the high rate of virus detected (94.7%) in this study underlined the various viral etiologies during the RSV epidemic season in very young infants with acute bronchiolitis. Dual RSV/RV infection was shown as the second cause of bronchiolitis, while RSV remained the first and, RV and hMPV the third and fourth respectively. The prevalence of RSV, RV and hMPV was indeed comparable to other studies [3], [5], [8], [16], [17], but we conversely found no difference in the age of the infants as regards the viruses involved. On the other hand, the seldom detection of parainfluenza virus or influenza A virus could be explained respectively by the recruitment restricted to the winter season and the very young age of the infants. The other viruses that were encountered were frequently found among various combinations of co-infection. Lastly, bocaviruses, prevalent viruses in acute bronchiolitis, were not researched in this series. However, its pathogenicity was to date uncertain, as it is mainly detected in coinfection [18].

We chose to enroll only hospitalized infants with a first acute bronchiolitis, with no underlying disease and no previous treatment in the way to respect the natural history. In this selected very young population, the distribution of the viruses allowed comparing infants with four different viral carriages: RSV alone, RV alone, hMPV alone and dual RSV/RV infection. Interestingly, the infants with RV had a less severe disease than those who carried either RSV alone or dual RSV/RV infection. Furthermore, they recovered quickly with a shorter stay in hospital. This result was contrary to the previously reported series from Papadopoulos *et al* [3]. These authors established that RV caused a 5.6 fold risk to be included in a high risk group defined by a clinical score above the 50^th^ percentile, and an earlier admission in the emergency unit. In our study, 56% of RSV infants and only 33% of RV had a score above this threshold and the infants with RV bronchiolitis presented at a later stage of the disease than the RSV infants. In addition, these authors found a difference in age and weight as regards the virus carried. Therefore, the discrepancies between both studies may likely be explained by the different population enrolled, and the clinical assessment of severity. More recently, Richard *et al.* [5] reported no difference in the distribution of RV or RSV among young infants hospitalized either in short term unit or PICU. Mansbach *et al.* [19] observed a better outcome for infants with RV bronchiolitis in Emergency Departments, and RSV was in other series identified as the more causative aggressive agent [12], [20]. Lastly, hMPV bronchiolitis featured a more initial hypoxemic disease without worsening the further outcome, as they differed in a shorter hospital stay from RSV but not from RV or RSV/RV illness. This associated hypoxemia was also observed by Mullins *et al.* [21] but not reported in other studies [4], [22].

The statement of viral co-infections in acute bronchiolitis raised the question of a cumulative pathogenic effect and consequently a more severe disease, although other hypotheses as successive infections or healthy carriage might be also supported [23]. In this study, the clinical severity of dual RSV/RV infection was comparable to that observed with RSV, but differed in a shorter duration of hospitalization in the coinfected infants. This underlined that RSV remains the most aggressive virus on the bronchi, and masked a clinical relevant additional effect of RV, in agreement with previous reports [3], [12], [20]. The other co-infections were not analyzed, because of the small number of infants in each category. Among the coinfections, the comparison of RSV and dual hMPV/RSV lower respiratory tract infection has been the most studied, and was associated with either no greater severity [16], [24] or an increased risk to undergo mechanical ventilation [25]. Richard *et al.* [5] also reported that any dual viral infection conferred a 2.7- fold increase in the risk to be admitted in PICU for ventilator assistance. This studied population was however characterized by a high proportion of premature newborns or history of underlying chronic illness and the assessment of the severity differed completely.

The young age has been established as one of the most relevant risk factors in RSV bronchiolitis. Commonly detected in infants younger than those who carried RV [3], [6] or hMPV [22], [26], RSV-associated severity was suggested therefore to be related to a younger age [6]. Although the age was strongly correlated with the duration of hospitalization, this hypothesis was not supported in our study, as the infants forming each viral subgroup were aged in a similar range. In addition, we found a slight increase of clinical severity in the girls, a longer time of hospitalization due to tobacco exposure and a protective effect of family history of atopy, which was also observed by Bradley *et al.* [27]. The multivariate analysis confirmed that the type of virus involved in the infection influenced independently the length of hospitalization. Other known virus-related risk factors of severity were not studied in this series, as well as the viral load [28], the host dependent [29] or virus-related inflammatory response [14].

In conclusion, the severity of a first acute episode of bronchiolitis in very young infants depends on the causative agents. Although RSV remains the main concern in this frequent respiratory illness, the prevalence of dual RSV/RV and RV infection is high and permitted to reconsider the use of corticosteroids [30]. However, proposing new management implies the routine use of RT molecular amplification, which is currently the only tool to easily target the detection of rhinoviruses, but would include an increasing cost in the investigations.
